# Automatic prediction of catalytic residues by modeling residue structural neighborhood

**DOI:** 10.1186/1471-2105-11-115

**Published:** 2010-03-03

**Authors:** Elisa Cilia, Andrea Passerini

**Affiliations:** 1 Information Engineering and Computer Science Department, via Sommarive 14 - I38100 (Povo) Trento, Italy

## Abstract

**Background:**

Prediction of catalytic residues is a major step in characterizing the function of enzymes. In its simpler formulation, the problem can be cast into a binary classification task at the residue level, by predicting whether the residue is directly involved in the catalytic process. The task is quite hard also when structural information is available, due to the rather wide range of roles a functional residue can play and to the large imbalance between the number of catalytic and non-catalytic residues.

**Results:**

We developed an effective representation of structural information by modeling spherical regions around candidate residues, and extracting statistics on the properties of their content such as physico-chemical properties, atomic density, flexibility, presence of water molecules. We trained an SVM classifier combining our features with sequence-based information and previously developed 3D features, and compared its performance with the most recent state-of-the-art approaches on different benchmark datasets. We further analyzed the discriminant power of the information provided by the presence of heterogens in the residue neighborhood.

**Conclusions:**

Our structure-based method achieves consistent improvements on all tested datasets over both sequence-based and structure-based state-of-the-art approaches. Structural neighborhood information is shown to be responsible for such results, and predicting the presence of nearby heterogens seems to be a promising direction for further improvements.

## Background

Discovering the molecular mechanisms underlying the protein functioning is a key step for understanding the complex processes involved in living systems, and would possibly allow to correct dysfunctions. Large scale genomics projects are providing a huge amount of protein sequential and, at a lower but increasing rate, structural information. Nonetheless, a large portion of such proteins have their function still undetermined, as it is often not straightforward to understand the details of a protein function even when its 3D structure is known. The task requires a time-consuming trial-and-error process of hypothesis formulation and verification by targeted experiments such as site-directed mutagenesis [1]. Considering the rate at which protein structures are solved, the gap with respect to functionally characterized proteins is destined to increase over time. Automatic approaches for the detection of protein functional sites can be very useful in narrowing this gap, by exactly determining functional residues or reducing the number of candidates to be experimentally verified.

In this paper we focus on predicting catalytic residues in enzymes. Enzymes are proteins able to accelerate chemical processes inside a cell. In the catalysis the enzyme works by forming complexes with the substrates, usually small molecules, and in doing so it lowers the activation energy of the reactions thus increasing their rate. According to their function enzymes are classified into six functional classes in the so called Enzyme Classification (EC) Nomenclature [2]. Many enzymes need to be bound to an additional non-protein component called cofactor in order to perform their function. Cofactors can be grouped in: (a) coenzymes, i.e. dissociable cofactors that are usually organic; and (b) prosthetic groups, i.e. non dissociable cofactors. The enzyme lacking the cofactor is inactive and it is called apoenzyme, while the enzyme with the cofactor is active and it is called holoenzyme. Enzymatic functional domains are also called active or catalytic sites. The residues that are directly involved in the catalytic process (e.g. nucleophiles, proton-donors) constitute the active site, while residues in the surrounding space play the role of attracting and orienting the molecule to bind, and constitute the binding domain. The first kind of residues are of interest in our study. From now on we refer to them as functional or catalytic residues. Traditional approaches to functional site identification use homology-based strategies. Novel protein function is inferred by aligning the sequences or by superimposing the structures with already annotated proteins. In [3], active sites of non annotated proteins in the Pfam database [4], which contains about 8,200 protein families, are predicted by using a rule-based technique which exploits the homology and sequence similarity with other annotated proteins. The methodology is based on the transfer of experimentally determined active site data to other sequences within the same Pfam family. The authors show that it is possible to gain functional annotation of a large number of sequences in the Pfam database (enzymatic families) for which the residues responsible for catalysis have not been determined. However, these homology-based techniques are well-known to fail in specific situations. First, an annotated homologue of the target protein needs to be available, preventing their applicability to novel folds. Furthermore, proteins with similar overall tertiary structure can have different active sites, i.e. different functions [5,6], and proteins with different overall tertiary structure can show the same function and similar active sites (an example on proteases can be found in [7]). Finally, the increasing lack of functional annotations makes transferring them by homology even less effective.

A number of researchers have recently tackled the problem of functional residues prediction. In [8] the authors generated three-dimensional templates of protein active sites with rigid prosthetic groups. Their approach is based on the simultaneous alignment of several protein structures, and relies on local atomic-level similarities based on multiple comparisons. The generated patterns include 3D atomic coordinates, position of chemical groups, and cavity locations. However the approach remains limited to the subset of proteins having rigid prosthetic groups.

In its simpler formulation, functional residue prediction can be cast into a binary classification task at the residue level. Petrova and Wu [9] and Youn et al. [10] have addressed it with a Support Vector Machine (SVM) fed with both protein sequence and structural properties. Capra and Singh [11] relied on an information-theoretic approach for estimating sequence conservation. The authors show that conservation of sequentially close residues improves predictive performance, especially when catalytic residues are involved. In [12], carefully crafted conservation scores were shown to play a major role in predictive performance. Closeness centrality measures [13] have been used in [14] to improve catalytic residue prediction by using neural networks trained with a genetic algorithm. A review on approaches and applications for structure-based protein function prediction can be found in [15]. Interestingly, a recent study [16] showed that sequence information alone could provide results similar to those obtained by previous structure-based methods. Our early experiments came to the same conclusions (see *Results*), with profile enriched sequential information providing results which were statistically indistinguishable from carefully crafted features extracted from 3D coordinates [9]. This seems to indicate that much work has still to be done in order to fully exploit the information contained in protein three-dimensional structures. A number of recent approaches investigated the use of topological [17], electrostatic [18] and graph theoretic [19] structured-based features for predicting ligand binding sites or protein functional sites. In a very recent work [20] electrostatic features from THEMATICS [21] and geometric features are combined with sequence conservation features in a maximum likelihood approach called Partial Order Optimum Likelihood (POOL). The authors underline the key role of THEMATICS features which are extracted from the residue theoretical titration curves.

In this work, we show how to effectively employ 3D structure information by modeling the structural neighborhood of candidate residues, represented as a sphere centered on the residue side chain. We encoded such neighborhood information with statistics on the properties of its content, such as physico-chemical properties, atomic density, flexibility, presence of water molecules. We trained a support vector machine combining our structural neighborhood features with evolutionary enriched sequence information as well as previously developed 3D features [9]. Our structure-based method achieves improvements over both sequence-based and structure-based state-of-the-art predictors, as measured on a set of benchmark datasets with varying characteristics, and structural neighborhood information is shown to be responsible for such improvements. We additionally investigated the role of ligand information in presence of heterogens, playing possible catalytic or structural roles, and showed that exploiting such information in both sequence-based and structure-based active site predictions is an interesting direction for further research.

## Results and Discussion

### Dataset

Functional residue prediction can be cast into a binary classification task at the residue level, namely predicting for each residue of a given protein, whether it is directly involved in the catalysis or not. We performed a detailed analysis and feature engineering on a dataset (PW) of 79 enzymes selected by Petrova and Wu [9] for their structural and functional heterogeneity with respect to their SCOP fold classification, EC numbers and BLAST sequence similarity. The dataset contains enzymes from all the six classes in the Enzyme Classification (EC) Nomenclature. We collected sequential and three-dimensional data for a total of 23,635 residues from the enzymes PDB files. Few residues were removed with respect to the 23,664 extracted in [9] due to uncertain correspondence in the mapping between the two datasets or due to conflicts between the residues reported in the PDB structure file and in the FASTA sequence from Uniprot [22]. Only 254 out of 23,635 residues are labeled as functional in the Catalytic Site Atlas (CSA) database [23]. Hence the dataset is strongly unbalanced (see Additional file 1) with a ratio between positive and negative examples of about 1:92.

We also conducted a broad experimental evaluation of the obtained features on a set of larger benchmark datasets which were proposed by previous sequence and structured-based approaches. Three benchmark datasets with varying homology level were proposed in [10]: a SCOP fold dataset (EF fold), a SCOP family dataset (EF family) and a SCOP superfamily dataset (EF superfamily). Two additional datasets were included to study the performance of our approach in the presence of low homology: the HA SCOP superfamily dataset from [13] and the independent test set T-124 proposed in [16]. The characteristics of these five datasets are summarized in [16]. Finally we included the dataset of 160 proteins (POOL-160) used in [20] in order to compare with their approach.

### Experimental Setting

We addressed the learning task with an SVM, a state-of-the-art binary classification algorithm which aims at separating positive and negative examples with a large margin, possibly accounting for margin errors. Details on SVMs can be found in several textbooks [24]. All experiments were carried out using the *SVM *^*Light *^[25] software [26] downloadable from http://svmlight.joachims.org/. Our experimental evaluation is based on a 10-fold cross-validation procedure stratified at the protein level, that is, assuring that all residues of a certain protein always appear together in the same fold.

We fixed the regularization parameter (parameter *c *in the *SVM *^*Light *^implementation) to 1, and tuned the cost factor (parameter *j *in the *SVM *^*Light *^implementation), which outweighs the error on positive examples with respect to that on negative ones, on each fold of the 10-fold cross-validation by an inner cross-validation procedure inside its training set. Tuning the cost factor is particularly important for this application due to the strong imbalance between the number of positive and negative examples. Previous works [14,16] addressed such a problem by subsampling negative examples according to a certain ratio and training the classifier on the reduced set.

#### Performance Measures

The following measures have been used to evaluate our approach:

• *Precision *= 

• *Recall *or *Sensitivity *or *TP rate *= 

• *FP rate (1-specificity) *= 

• *F*_1 _*measure*, *F*_1 _= 

• *Matthews Correlation Coefficient *= 

• *Area Under the Averaged Receiver Operator Characteristic (ROC) Curve *(AUCROC)

• *Area Under the Averaged Recall/Precision Curve *(AUCRP)

where *t*^+^, *t*^-^, *f *^+^, *f *^-^are the true positives, true negatives, false positives and false negatives respectively.

*F*_1 _is the harmonic mean between *Recall *and *Precision*, giving equal weight to the two complementary measures. It is the measure we optimized in our model selection phase. The averaged ROC and RP curves are drawn by averaging the per-protein curves as in [20]. ROC and RP curves and their areas provide a broader picture of a classifier performance, as they do not require to choose a fixed decision threshold to discriminate positive and negative examples, but evaluate all possible thresholds. For highly skewed datasets, the area under the RP curve is more informative than the area under the ROC [27]. We included both measures to allow for comparisons with previous approaches.

#### Statistical Tests

We evaluated the statistical significance of the performance differences between the various settings by paired Wilcoxon tests on the *F*_1 _measure reported for each fold. We employed a confidence level *α *of 0.05.

### Overview of the extracted features

We extracted different sets of features from both primary and tertiary protein structure in order to represent candidate residues. Tables 1 and 2 summarize our sequence and structural features respectively. In the following we give a brief overview of such features, while a detailed description is provided in the *Methods *section.

**Table 1:** Sequence-based features.

Features	Description
1*D*_1_	Target amino acid name
1*D*_2_	Target amino acid type
1*D*_3_	Conservation profiles

Representation: features extracted from the protein sequence.

**Table 2:** Structured-based features.

Features	Description
3*D*_1_	Physical and chemical properties (amino acid attributes)
3*D*_2_	Amino acidic composition
3*D*_3_	Charge/Neutrality
3*D*_4_	Water molecule quantity
3*D*_5_	Atomic density
3*D*_6_	Flexibility B-factor

3*D*_7_	Disulphide bond
3*D*_8_	Heterogens
3D_9_	Cofactor binding

Representation: scalar features extracted from the residue structural neighborhood.

From the protein sequence we extracted a conservation profile (1*D*_3_) capturing evolutionary information, together with standard attributes encoding the name and type of the amino acid (1*D*_1 _and 1*D*_2_).

From the protein tertiary structure we extracted features characterizing the three-dimensional neighborhood of a residue: statistics on the neighborhood properties such as physico-chemical characteristics (3*D*_1_), type and frequencies of the neighboring amino acids (3*D*_2_), charge or neutrality of the surrounding space (3*D*_3_), presence of the water (3*D*_4_), atomic density (3*D*_5_), flexibility of the target residue (3*D*_6_); the presence of disulphide bridges involving the target residue (3*D*_7_); information on the presence of potential cofactors or other ligands, by encoding the presence of nearby heterogens playing possible catalytic or structural roles (3*D*_8_) as well as the fact that they directly bind the target residue (3*D*_9_). The *Methods *section gives a detailed explanation of such structural features and the rationale behind their use.

Our aim was to exploit information related to the properties of the local structure surrounding a residue. We added these features to those already used in [9], which aim at modeling properties of the target residue plus its relationship with the whole region containing it. Such combined representation allowed us to obtain significant improvements, as detailed in the *Results *section.

Table 3 reports a legend of the abbreviations we employed for the different sets of attributes that we tried. These sets of features include also the set of 24 attributes proposed in [9].

**Table 3:** Legend of abbreviations.

Abbreviation	Description
*SVM_P*5_1*D*_	the attributes extracted from the protein sequence among the 24 in [9]
*SVM_P*_24_	the whole set of 24 attributes proposed in [9]
*SVM_P*_7_	the optimal set of 7 attributes selected among the 24 in [9]
*SVM_*1*D*_*i*-*j*_, 3*D*_*k*-*r*_	the attributes from 1*D*_*i *_to 1*D*_*j *_and/or from 3*D*_*k *_to 3*D*_*r *_as described in section *Methods*, with *i, j *= 1, 2, 3 and *k, r *= 1, ..., 9

Legend of abbreviations for the different sets of attributes tried in the experiments (see *Methods*).

### Results of different feature sets

We conducted a set of experiments aimed at elucidating the role of the different feature sets on the PW dataset (see Additional file 2 for the 10-folds used in the experiments on the PW dataset). Preliminary experiments showed that polynomial (second and third degree) or Gaussian kernels did not significantly improve performance with respect to simpler linear kernels. All reported results thus refer to the latter type of kernel. Table 4 reports a summary of experimental comparisons for different sets of sequence- and structure-based features we used.

**Table 4:** Feature evaluation.

		**Performance % ± *s.d***.						
	**CV Exp**	**P**	**R**	**FPR**	***F*_1_**	***MCC***	***AUCROC***	***AUCRP***

1.	*SVM_*1*D*_1-3_	22 ± 11	30 ± 11	1.3 ± 0.7	24 ± 7	24 ± 8	0.9172	0.2777
2.	*SVM_P*5_1*D*_	26 ± 8	29 ± 12	0.9 ± 0.3	27 ± 9	26 ± 9	0.9311	0.3129
3.	*SVM_P*5_1*D*__1*D*_1-3_	27 ± 10	30 ± 10	1.0 ± 0.4	27 ± 8	27 ± 8	0.9370	0.3204

4.	*SVM_P*7	22 ± 11	37 ± 11	1.8 ± 1.3	26 ± 10	27 ± 10	0.9490	0.3532
5.	*SVM_P*24	26 ± 10	37 ± 14	1.2 ± 0.5	30 ± 9	30 ± 10	0.9529	0.3605
6.	*SVM_P*24_1*D*_1-3_	26 ± 6	44 ± 10	1.4 ± 0.3	32 ± 7	33 ± 7	0.9556	0.3659
7.	*SV_M P*24_1*D*_1-3_, 3*D*_1-6_	28 ± 9	46 ± 10	1.4 ± 0.5	34 ± 8	34 ± 8	0.9635	0.3723

8.	*SVM_P*24_1*D*_1-3_, 3*D*_1-9_	33 ± 14	48 ± 8	1.4 ± 0.7	37 ± 7	38 ± 6	0.9633	0.4125

Summary of the results of the cross-validation on different selected attributes (linear kernel, regularization parameter *c *= 1).

The first set of experiments refers to a sequence-based functional residue predictor, where each residue is characterized by features extracted from the protein sequence only (see Table 1). In Table 4, row 1 reports experimental results obtained by using our sequence-based attributes only, including the multiple alignment conservation profiles. We also experimented windows of conservation profiles of size varying between 1 and 10, where size *w *implies a window of *w *residues on each side of the target residue along the primary sequence, in addition to the profile of the target residue itself. Including such windows only provides a slight improvement (with *w *= 7) while drastically reducing the classifier efficiency. Furthermore, the features proved harmful when combined with structural information, possibly because the large number of features they introduced covered the signal coming from other more informative ones.

Rows 2 and 3 report additional results on sets of attributes extracted from sequence information only. The set *SVM_P*5_1*D *_is a group of five attributes from [9] which includes the 1*D*_1 _and 1*D*_2 _attributes (see Table 1) and a conservation score from the Scorecons server [28], plus its entropy and relative entropy values, in place of our conservation profile. The results are comparable with those obtained with conservation profiles. Results combining all the available features extracted from the protein sequence are reported in row 3 (*SVM_P*5_1*D*__1*D*_1-3_).

Results in the rows from the fourth on include additional information provided by structural features. In rows 4 and 5 we employed the two sets of attributes proposed in [9], i.e. the subset of the 7 optimal ones (*SVM*_*P*7) and the entire set of 24 attributes (*SVM*_*P*24) respectively. Note that we obtained performance improvements over the original results in [9] (achieving *F*_1 _= 13% and MCC = 23% for the *P*24 feature set) by tuning the cost factor for false positives versus false negatives, as compared to random sub-sampling negative examples in order to obtain a balanced set.

Table 5 reports F_1 _measures of our best combination of sequence-based features, the sequence and structure based features from [9], plus our additional set of structural neighborhood features, excluding those coming from ligand information. Results are reported for all test datasets described in the *Datasets *section. The first relevant finding is that appropriate sequence-based features taking into account evolutionary information (*SVM_P*5_1*D*_*_*1*D*_1-3_) achieve performance which are comparable to carefully crafted structure-based ones [9] (*SVM_P*24). The difference is never statistically significant in all tested datasets. This confirms the finding of [16] that state-of-the-art sequence-based predictors have performance comparable with recent structured-based approaches. Selecting the appropriate and discriminant structural attributes for functional residue prediction is thus not a trivial task.

**Table 5:** Statistical analysis.

	**HA superfamily**	**EF fold**	**EF superfamily**	**EF family**	**PW**	**POOL-160**
*SVM_P*5_1*D*_1-3_	23	24	25	24	27	24
*SVM_P*24	21	24	24	23	30	23
*SVM_P*24_1*D*_1-3_, 3*D*_1-6_	26 ◦ ∙	28 ◦ ∙	27 ◦ ∙	28 ◦ ∙	34 ◦	27 ◦ ∙

Statistical comparisons of our best set of sequence-based features (*SVM_P*5_1*D*_1-3_), the set of sequence-and structure-based features employed in Petrova and Wu [9] (*SVM*_*P*24), and their combination with our additional set of structural neighborhood features (*SVM_P *24_1*D*_1-3_, 3*D*_1-6_), excluding those coming from ligand information. Cross-validated *F*_1_measures (%) and results of a paired Wilcoxon test (*α *= 0.05) on the statistical significance of the performance differences are reported for all benchmark datasets employed in this study. A white circle indicates a statistically significant improvement of the classifier in the row over the sequence-based classifier (*SVM_P*5_1*D*_1-3_), while a black bullet indicates a statistical significant improvement over the Petrova and Wu features (*SVM_P*24).

On the one hand, using features extracted from primary sequence alone allows us to apply the predictor to the much larger set of sequentially but not necessarily structurally determined proteins. On the other hand, as we already discussed in the introduction and also stated in the review of [15], the availability of structural information should be able to significantly contribute in solving the task. Indeed, adding three-dimensional information in the form of properties of the residue structural neighborhood allowed us to achieve significant improvements, as detailed below.

Row 6 in Table 4 reports results of the combination of our conservation profiles (1*D*_3_) with all the sequence and structural attributes in [9]. Row 7 reports the result obtained by adding structural attributes encoding statistics of the residue three-dimensional neighborhood properties (3*D*_1-6_) without including the attributes related to the ligands (3*D*_7-9_, see *Methods*). Such results are always significantly better than those of sequence-based classifiers according to the statistical tests (see Table 5). Furthermore, performance improvements with respect to previous structure-based results (*SVM_P*24) are significant in all but the smallest test set.

Finally, row 8 reports the performance obtained by including all the available ligand-based features, which allow to achieve further improvements and correctly predict some especially tough cases (detailed below), paving the way to an interesting research direction. Additional files 3 and 4 report detailed results and predictions for this classifier.

**Figure 1:**
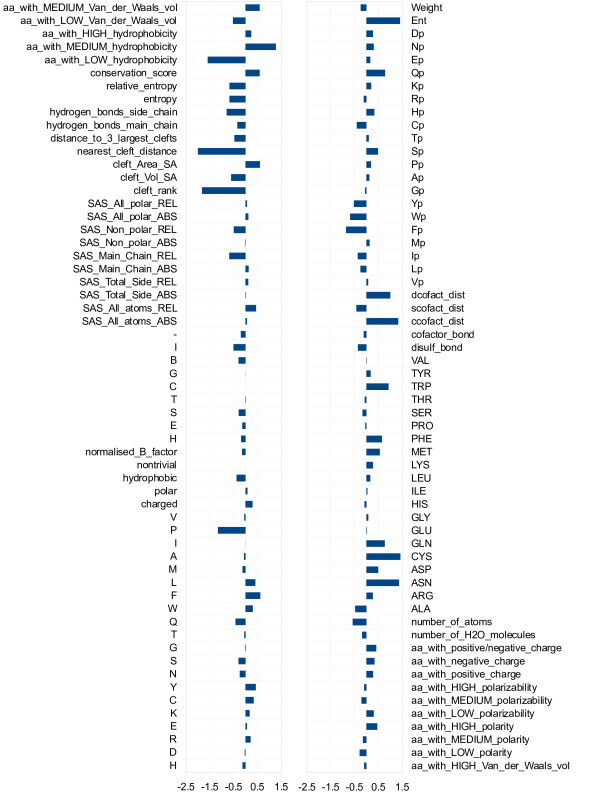
**Feature weight vector**. Vector of the feature weights  of a classifier trained on the PW dataset.

In order to better understand which are the features contributing the most to the classifier performance, we analyzed the weight vector  describing the separating hyperplane learned by the SVM. The  components with higher absolute value are associated to the most discriminant features of the classifier. In Figure 1 we represent the weight vector of a classifier trained on the PW dataset. Among the most relevant properties there are features related to the relative position on the protein surface (*cleft_rank*, *cleft_Vol_SA*, *cleft_Area_SA*, *nearest_cleft_distance*), features related to the conservation along the primary sequence (*conservation_score*, *entropy *and *relative_entropy *plus some other features from the conservation profiles 1*D*_3_) and also features describing the structural residue neighborhood. For instance, the fractions of amino acids with low and medium hydrophobicity are quite discriminative in opposite directions. The same holds for low and medium Van der Waals volume. Other discriminative features include the atomic density of the residue sphere and the features related to its amino acidic composition: in particular the number of ASN, CYS and GLN residues, but also that of MET, PHE and TRP ones.

Further analyses on the effect of different sets of features on prediction errors provide some interesting insights on their usefulness and reliability. The quality of multiple alignments strongly influences the performance of sequence-based classifiers. On the proteins for which PSI Blast did not provide good alignments we observed poor performance. In those cases structural features help in compensating such deficiencies. The inclusion of ligand features allows the correct prediction of many catalytic residues which have low catalytic propensity, like the glycine in the methylglyoxal synthase (PDB code 1B93) and the glycine in the human glutathione synthetase (PDB code 2HGS). The latter is one of the emblematic cases of the importance of ligand features, as in the absence of those features only one of its four catalytic residues is correctly predicted. In the phosphofructokinase (PDB code 1PFK) the encoding of ligand features helps to correctly predict the two arginine residues of the active site. By looking at the three-dimensional structure of the protein, the active site seems to be exposed rather than located in a hydrophobic core. This implies that active site residues have associated structural features which may differ from those typical of the other catalytic residues in the dataset. On the other hand, we also observed few cases in which the addition of ligand features worsens predictions. This happens mainly when no heterogen appears in the crystal structure, possibly because the enzyme was solved in its apo form. We are planning to verify such conjecture by applying techniques for detecting binding sites in 3D structures [29]. Note that while the presence of a heterogen provides a clear hint that the area could contain an active site, it is not by itself sufficient to determine the set of catalytic residues. Out of the 365 heterogen-binding residues in the dataset, only 62 were actually labelled as catalytic. If we restrict to the subset of heterogens which tend to occur near catalytic sites in enzymes (see the *Ligand features *section), the fraction becomes 51 out of 285. As detailed in [30], the sole fact of binding a substrate or cofactor does not classify a residue as catalytic. It also has to perform some specific activity such as proton abstraction from substrate, cofactor or water activation. For instance, the above-mentioned phosphofructokinase (PDB code 1PFK) contains three heterogens: ADP, beta-fructose diphosphate (FBP), and a magnesium ion; of the 15 residues which bind one of them, only four are actually catalytic. In this case the predictor manages to selectively exploit ligand information in identifying two active arginine residues, one of which does not directly bind any heterogen, with a single additional FBP-bound arginine incorrectly predicted as catalytic. Given that information on binding residues helps detecting active ones, it would be interesting to predict it when missing, either because sequence information alone is available, or because the 3D-structure does not contain the bound cofactor and/or substrate. Indeed, both binding and active residues should be identified in order to fully characterize the functional domain. We believe that combining active and binding site prediction in a single collective model, as already done with profile-HMM for specific functional domains [31], is a promising research direction, which can rely on a number of works for predicting binding sites from both sequence [32,33] and structural information [29,34].

#### 3D kernel

In order to further investigate the discriminative potential of the features extracted from the 3D residue neighborhood we also experimented a structured kernel. We employed a 3D decomposition kernel on planar shapes in the 3D space. This kernel was proposed in [35] for the classification of small molecules. We adapted it to the functional residue prediction task by extracting specific shapes from the residue structural neighborhood. Among the different design choices we tried, the best performing one was the set of planar shapes of two (segment) and three (triangle) vertices in the 3D neighborhood of a residue. One of the vertices was the target residue itself, and the others were residues evolutionary conserved over one of the hydrophobic, charged or polar classes. While providing reasonable performance when used alone, with an average *F*_1 _of 22% and an average MCC of 25%, such shapes failed to improve performance in combination with the remaining sequence- and structure-based features. This result confirms that effectively exploiting three-dimensional information for modeling catalytic residues is a hard task, and further research is needed.

### Comparison with other methods

We conducted a broad range of experiments on multiple benchmark datasets (see the *Dataset *section), and compared our results with the most recent methods for both sequence-based and structure-based prediction. Considered that none of the other methods directly encodes information on heterogens, we excluded such features from our set in all these comparisons.

**Figure 2:**
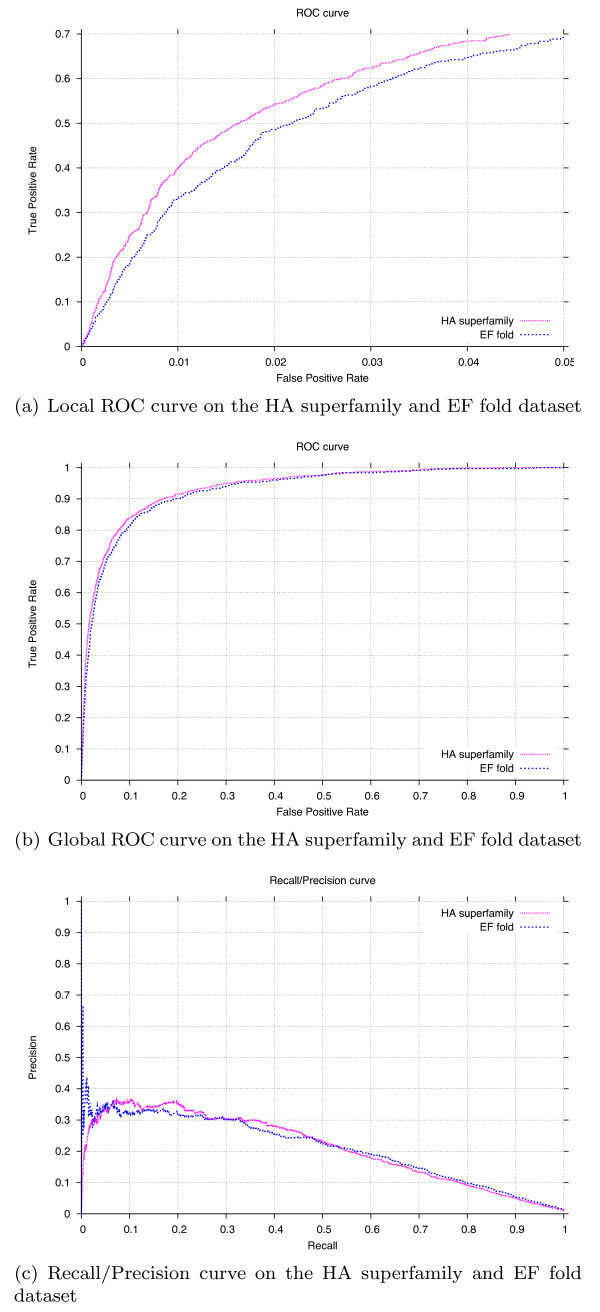
**ROC and Recall/Precision curves**. ROC and Recall/Precision curves of the predictions on two low homology benchmark datasets.

Table 6 shows experimental comparisons with the state-of-the-art sequence based predictor CRpred [16] on a number of datasets. Adhering to the setting in [16], we employed a 10-fold cross-validation procedure for all datasets but the T-124 one, for which we trained a single predictor on the entire EF fold dataset and tested it on the T-124 one. Our structural neighborhood features allow to consistently improve performance on all datasets, as measured by recall at equal precision, and precision at equal recall. The ROC and RP curves for the two low homology datasets HA superfamily and EF fold are shown in Figure 2, while those for the other datasets are available in the Additional file 5.

**Table 6:** Comparison with state-of-the-art sequence-based approach [16].

**Method**	**Datasets of competing methods**						
		
		**HA superfamily **[13]	**EF fold **[10]	**EF superfamily **[10]	**EF family **[10]	**PW **[9]	**T-124 **[16]
		
CRpred	R	54.0	48.2	52.1	58.3	53.7	50.1
	P	14.9	17.0	17.0	18.6	17.5	14.7
		
				*SVM_P*24_1*D*_1-3_, 3*D*_1-6_			
		
Equal P	R	67.4	64.6	66.2	61.3	69.7	54.8
Equal R	P	21.0	24.1	23.9	20.5	22.5	15.5

Comparison with the CRpred [16] sequence-based approach on six benchmark datasets. For each dataset we report recall obtained by our predictor at a precision equal to that of the competing method and precision at equal recall. Results are obtained without including ligand information.

Previous results [16] suggested that appropriate sequence-based features managed to match performance of different structure-based predictors on the same datasets, a result we also observed in our early experiments on the PW dataset. Conversely, the improvements we achieve here show that structural information can indeed be effectively employed in predictions. Nonetheless, further research is needed in order to fully exploit it, as our results using heterogen information seem to indicate.

Table 7 reports comparisons with the structure-based predictors from Chea et al. [13] and Youn et al. [10] for each of the benchmark datasets. Results, again measuring recall at equal precision and precision at equal recall, clearly indicate that our structural features consistently improve over the different methods on all datasets.

**Table 7:** Comparison with the structure-based approaches by *Chea et al*. [13] and *Youn et al*. [10] on their benchmark datasets.

**Method**	**Datasets of competing methods**				
		
		**HA superfamily **[13]	**EF fold **[10]	**EF superfamily **[10]	**EF family **[10]
		
Competing methods	R	29.3	51.1	53.9	57.0
	P	16.5	17.1	16.9	18.5
		
			*SVM_P*24_1*D*_1-3_, 3*D*_1-6_		
		
Equal P	R	63.4	64.2	67.3	61.7
Equal R	P	30.9	22.1	22.5	20.9

For each dataset we report the recall obtained by our predictor at a precision equal to that of the competing method and the precision at equal recall. Results are obtained without including ligand information.

Table 8 reports experimental comparisons with an additional structure-based predictor recently developed by Tang et al. [14] and tested on the PW dataset: the GANN method employs a neural network trained using a genetic algorithm. It includes a highly discriminant feature measuring network centrality, which accounts for the tendency of catalytic residues to have multiple interactions with other residues. The *F*_1 _and MCC measures of the two methods do not allow to draw clear conclusions, with our method achieving better *F*_1 _and worse MCC with respect to GANN. However, the availability of the detailed predictions of the cross-validation allows us to evaluate the overall threshold independent performance by areas under the ROC and RP curves. Both of them clearly show the advantages of our structural features.

**Table 8:** Comparison with the structure-based approach by *Tang et al*. [14] on the PW dataset.

	Performance %						
**Method**	**P**	**R**	**FPR**	***F***_1_	***MCC***	***AUCROC***	***AUCRP***

*Tang et al. (GANN)*^1^	19^2^	73	3.8	31^2^	36	0.9313	0.3556
*SVM_P*24_1*D*_1-3_, 3*D*_1-6_	28	46	1.4	34	34	0.9635	0.3723

^1^subsampling of negative examples with a ratio of 1:6 w.r.t. positives

^2^directly computed

Results include both performance measures at fixed decision threshold and average areas under ROC and RP curves. Results are obtained without including ligand information.

**Figure 3:**
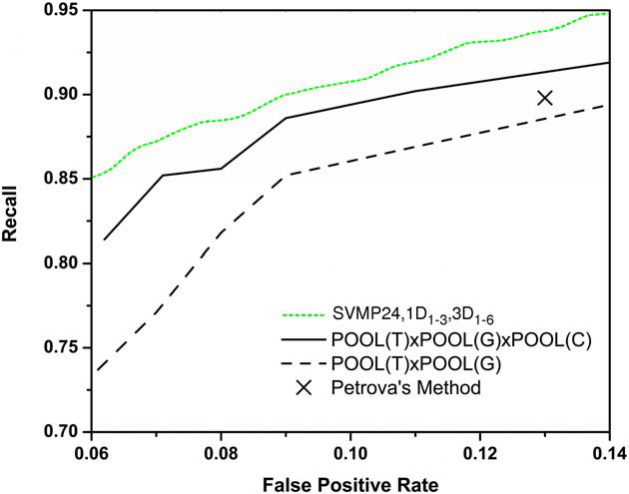
**ROC curves for comparison with Tong et al., 2009**. ROC curves superimposed with those reported in [20] on the PW dataset.

Finally, we compared with the recent structure-based predictor POOL [20] (Partial Order Optimum Likelihood), which combines effective electrostatic features from THEMATICS [21] with geometric and sequence conservation features in a maximum likelihood approach. Averaged ROC curves are reported in Figure 3. We compared our method and the POOL predictor with different sets of features as taken from [20]. The point representing Petrova and Wu results was also included in the graph. Our method achieves superior recall for all possible values of the false positive rate. We also conducted experiments on the dataset of 160 proteins proposed by the POOL authors [20]. In Table 9 we compare our results with the results of the best classifier (*POOL*(*T*) × *POOL*(*G*) × *P OOL*(*C*)) reported in [20] at equal recall and at equal precision. Averaged Recall (AvgR) and Precision (AvgP) are computed as in [20] as averages at the protein level. The area under our averaged ROC curve is 0.9523 as compared to 0.925 achieved by the best set of features for POOL.

**Table 9:** Comparison with the best results reported for the POOL structured-based method [20] on their benchmark dataset of 160 proteins.

		Performance %		
	**Method/Dataset**	**AvgP**	**AvgR**	***AUCROC***

1.	POOL(T)POOL(G)POOL(C)/allprotein (*Tong et al*. [20])	19.07	64.68	0.925
2.	*SVM_P *24_1*D*_1-3_, 3*D*_1-6 _at Equal Precision	19.07	78.10	0.948
3.	*SVM_P *24_1*D*_1-3_, 3*D*_1-6 _at Equal Recall	26.61	64.68	0.948

Performance measures include: the average per-protein precision at equal recall, the average recall at equal precision, and the average area under the ROC curve (AUCROC). Results are obtained without including ligand information.

## Conclusions

In this work we addressed the problem of predicting catalytic residues from protein sequence and structure. We developed an effective approach to exploit structural information, by modeling residue structural neighborhood as a spherical region centered on the side chain centroid and including various statistics on the properties of the neighborhood content. Our method outperforms the current state-of-the-art sequence-based and structure-based approaches, as shown on different benchmarking datasets. We further explored the information provided by the presence of nearby heterogens, playing possible catalytic or structural roles, when such information is available from the solved structure. We showed that ligand information can play a key role in correctly identifying functional residues with low catalytic propensities, and we are currently investigating solutions to jointly predict active and binding residues in the site in a fully collective approach.

## Methods

### Features Extracted from the Sequence

The features extracted from the primary sequence encode characteristics of the target residues and evolutionary information (see Table 1):

1*D*_1 _encodes the amino acid name of the residue.

1*D*_2 _encodes the amino acid type of the residue based on its physico-chemical properties: H, R, K, E, D as charged; Q, T, S, N, C, Y, W as polar and G, F, L, M, A, I, P, V as hydrophobic [30].

1*D*_3 _encodes evolutionary information in the form of multiple alignment profiles.

1*D*_1 _and 1*D*_2 _are categorical (or nominal) attributes, and are encoded one-hot: each attribute is encoded with a vector of bits of size equal to the number of possible attribute values; value *k *is encoded with a vector having one at position *k*, and zero at all other positions. 1*D*_3 _is a real vector of conservation profiles computed from multiple alignments. We performed a two iteration Position-Specific Iterative Blast Search (PSI-Blast) [36] on a database of non-redundant protein sequences (nr) downloadable from ftp://ftp.ncbi.nlm.nih.gov/blast/db/. A threshold of 5e-3 on the expectation value was employed for both initial iteration and extending hits. We enriched the profile extracted from the multiple alignment with two values indicating its informativeness and reliability, namely profile entropy and weight of the conservation profile with respect to pseudocounts.

### Features Extracted from the Structure

#### Residue Structural Neighborhood

**Figure 4:**
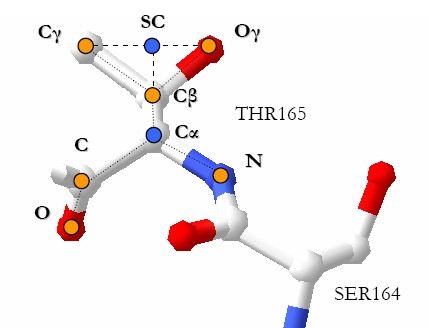
**Centroids**. A residue 3D representation: point SC is the side-chain centroid, which we used as the residue representative point.

We represent a residue in the 3D space as a single representative point, the centroid of its side-chain atoms (point SC in Figure 4), since such atoms are more likely to be involved in the catalysis. The single representative point of a glycine residue is the carbon-alpha (*C*_α_) atom.

Given such a 3D representation of residues, we define the structural neighborhood of a residue *x *as the set of residues and molecules contained in the volume of a sphere centered on *x *(*x *will be a target residue in our setting).

One can consider spherical regions of different radius. In this work we fix the radius of the sphere to a maximum of 8 Å which is the maximum interaction distance between a residue and a water molecule. The rationale behind this choice is that the interaction with a water molecule is very important for the catalysis in enzymes like the hydrolases.

**Figure 5:**
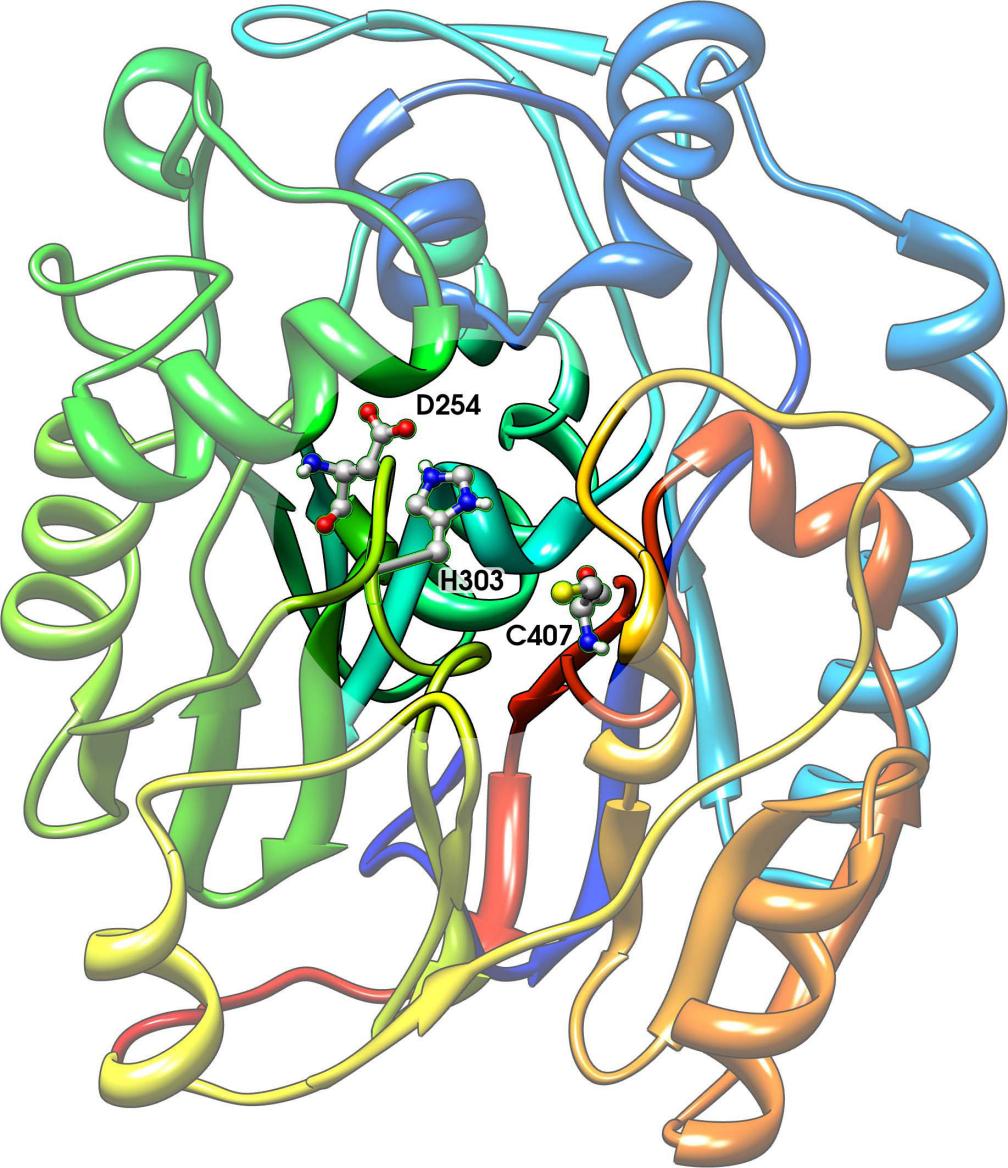
**Active site of L-arginine:glycine amidinotransferase**. The L-arginine:glycine amidinotransferase (1JDW) and its highlighted catalytic pocket.

**Figure 6:**
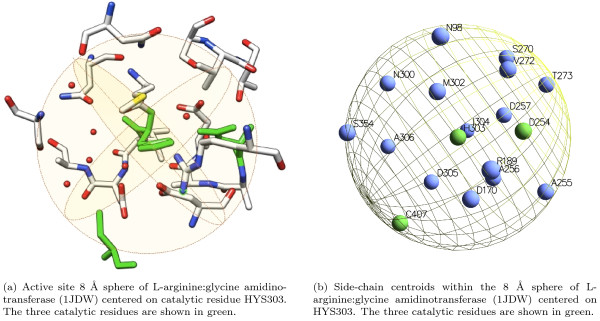
**Structural neighborhood**. A residue structural neighborhood.

As an example, Figure 5 shows the crystal structure of L-arginine glycine amidinotransferase (PDB code 1JDW), a mitochondrial enzyme involved in the creatine biosynthesis. The catalytic pocket is highlighted and the catalytic triad of residues is shown: ASP254, CYS407, HIS303 [1]. The cysteine is the nucleophile and binds the carbon on the substrate (arginine) side chain. The histidine activates the substrate to deprotonate CYS407 and deprotonates glycine, while the aspartic acid primes the histidine by activating water, a cofactor or a residue. In Figure 6(a) we show the 8 Å sphere centered on the HYS303 residue: the sphere contains all active site residues (shown in green). In Figure 6(b) we show the same sphere with residues represented with their side-chain centroids.

#### Structure-based Features

Features characterizing a residue can be extracted from the protein structure if available. We showed (see *Results*) that extracting features from a residue neighborhood, thus exploiting the locality of the protein structure, can be useful to discriminate between functional and non functional residues. Table 2 summarizes the scalar features we extracted from the residue 3D neighborhood. The first group contains statistics on the properties of the neighborhood content, while the second encodes information on possible ligands contained in the neighborhood. Each row in the table corresponds to an attribute or a set of attributes encoding the properties specified in the description. In the following we provide a detailed description of such features. The paragraph ends with a description of the 3D shapes we extracted from the structural neighborhood, which proved reasonably informative when applied alone, but failed to improve the results in combination to the other features, as discussed in the *Results *section.

**Figure 7:**
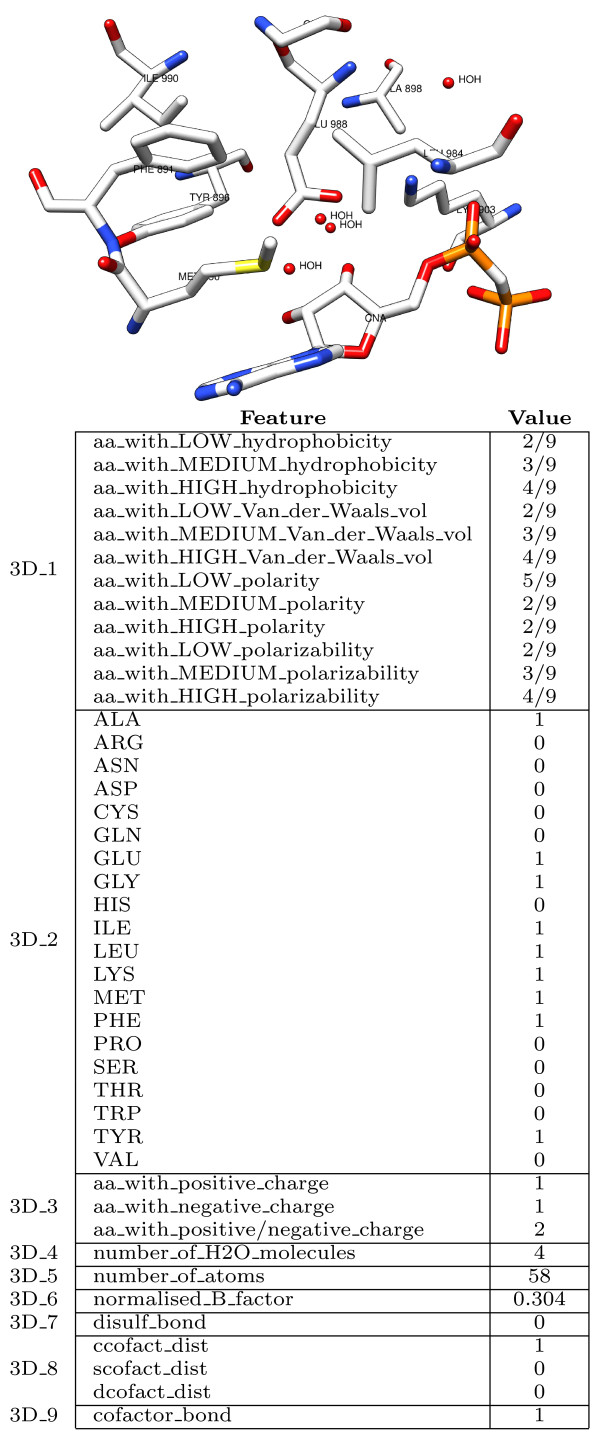
**The 3D feature vector**. An example of feature vector extracted from the three-dimensional neighborhood of the (catalytic) residue GLU 988 in the poly(adp-ribose) polymerase (1A26).

In Figure 7 we provide an example of feature vector extracted from the 3D-structural neighborhood of the target residue (GLU 988 of the PDB protein structure 1A26).

#### Statistics of the Neighborhood Properties

The first set of features encodes aggregate values representing properties of the atoms included in the sphere.

3*D*_1 _encodes chemical and physical properties of the residue neighborhood. This set of attributes represents properties such as hydrophobicity, polarity, polarizability and Van der Waals volume of the neighboring residues. They are encoded in a three bin distribution (normalized number of residues with low, medium, high hydrophobicity, polarity, polarizability and Van der Waals volume) according to the indices reported in the Amino Acid Index Database [37]. The same encoding was used in [38] for protein function classification.

3*D*_2 _encodes the amino acid composition of the 3D sphere, represented as the frequency of occurrence of each one of the twenty amino acids.

3*D*_3 _represents charge or neutrality of the 3D sphere, encoded into three values: the number of positively charged residues, the number of negatively charged residues and their sum.

3*D*_4 _encodes the quantity of water in the sphere, measured as the number of water molecules within the sphere radius. This group of attributes is motivated by the fact that an active site is usually located in a hydrophobic core of the protein, while on the surface the quantity of water is higher and the residues exposed to the solvent are not hydrophobic.

3*D*_5 _measures the atomic density of the sphere, calculated as the total number of atoms it contains.

3*D*_6 _represents the residue temperature factor (B-factor), as a measure of the residue flexibility. It is calculated as the average of the atomic B-factors of atoms composing the residue, normalized over the whole protein. As the temperature factor could depend on the crystal structure, normalizing over the whole protein helps to exclude the variations that can be present among different protein crystal structures. Note that in [9] an unnormalized version of the residue B-factor was employed instead.

#### Ligand Features

In oxidizing environments, cysteines tend to form covalent bonds called disulphide bridges, which help stabilizing the 3D structure of the protein. Disulphide bonded cysteines are usually not involved in the catalytic process: in the PW dataset of 79 enzymes the only exception is given by a protein disulphide isomerase (PDB code 1MEK). It has two catalytic cysteine residues in a thioredoxin domain similar to one of the well-known thioredoxin proteins. We encoded information on bridges by a flag (3*D*_7_) indicating whether the target residue is a disulphide bonded cysteine.

Enzymes often employ cofactors in order to help interacting with the substrate. Therefore, the presence of a cofactor in the structural neighborhood of a certain residue is an indication that the area could be an active site. On the other hand, many heterogens bind residues for structural rather than catalytic purposes, like NI in the methylmalonyl coa decarboxylase (PDB code 1EF8) [39] which is involved in trimerization. The Het-PDB Navi database [40] provides information on a large set of small molecules found in the protein structures of the PDB. For example information about the reaction in which the cofactors, substrates and products are involved, and the cofactor interface propensity. A description of the mechanisms of the catalysis is included in the CSA functional annotations whenever such information is available. It describes the role of the cofactors and which are the substrates and products of the reaction. In the dataset that we used for the feature engineering, 51 out of 79 enzyme structures contain heterogen molecules. For the remaining structures we can not say whether they are apoenzymes or they just do not require any help from cofactors during the catalysis. In the former case, methods for predicting metal-binding sites in apo protein structures [29] may be used to identify the presence of possible cofactors.

**Figure 8:**
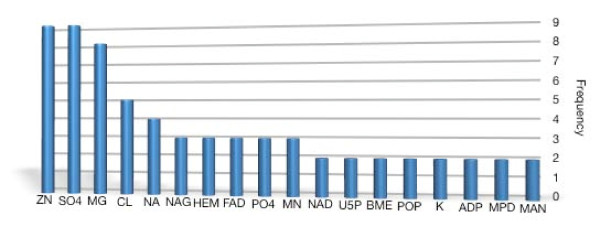
**Frequency of the Heterogens in the Dataset**. Histogram of the frequencies of heterogen molecules in the PW dataset (79 enzymes). Only the heterogens appearing in more than one protein structure are reported.

In Figure 8 we show a histogram of the most frequent heterogens we found in the PW dataset. Each one of those heterogens appears at least in two protein structures. All the details about the heterogens and their 3 letter code in PDB can be found in the Het-PDB Navi database [41].

According to our analysis on this set of proteins, most of those heterogens have a demonstrated or putative role in the catalytic process (ZN, NAG, NAD, BME, MG, MN, U5P, ADP, HEM, FAD, MPD), while for others this role can be clearly ruled out (CL, NA, K, MAN), or it is just uncertain (PO4, SO4, POP). In order to correctly encode discriminant features related to the presence of cofactors, we divided the heterogen molecules into groups (at least the 71 we found in the PDB dataset, excluding DNA molecules) based on their physico-chemical, functional, spatial or shape characteristics.

As an example, in this dataset of 79 enzymes ZN usually has a verified role within the active site, thus we considered it as a primarily catalytic cofactor. Actually among the whole set of known enzymes there are cases, such as the DNA glycosylase, having a zinc-finger in which ZN has a structural role. We believe other features of the residue 3D neighborhood (e.g. four cysteine residues in the same sphere around a ZN atom) should help discriminating functional from non functional residues in these cases.

**Figure 9:**
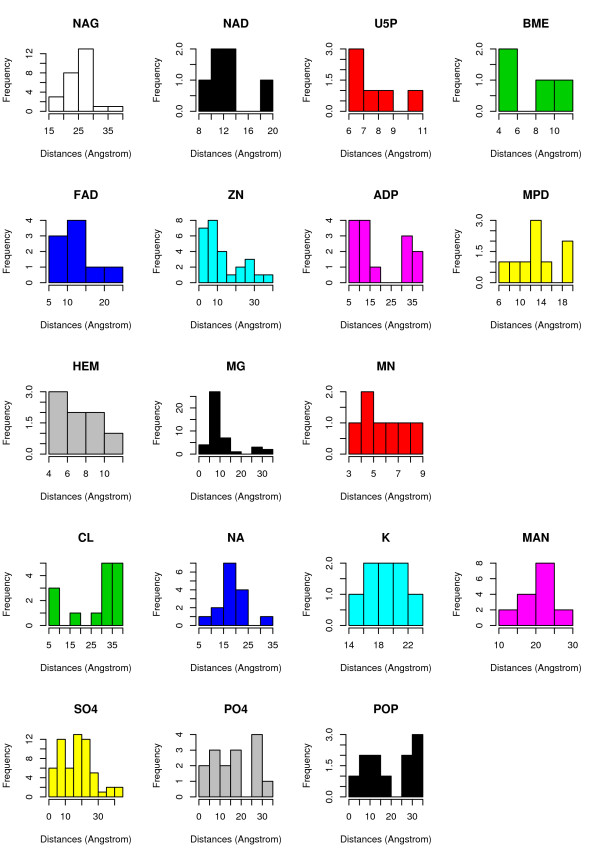
**Heterogens Distances from Catalytic Residues**. Histograms of the distances of the most frequent heterogens from catalytic residues in the PW dataset.

We analyzed the distances of the heterogens from the catalytic residues, representing each heterogen by the centroid of the atoms composing it. We observed that the role mentioned in the literature is correctly reflected by the distribution of the distances from the catalytic residues. Figure 9 reports histograms of the distance of each one of the most frequent heterogen from catalytic residues. The first three rows contain heterogens having a role in the catalytic site: the peak of the frequencies is around values between 3 Å and about 15 Å depending on the space occupancy of the molecule. One exception is given by the N-acetyl-D-glucosamine (NAG) which is a monosaccharide that takes part in enzymatic processes like glycosilation: its average distance from the protein will make its presence in the residues neighborhood quite a rare event. The fourth row of histograms relates to non-catalytic heterogens: the frequency peak is shifted around values greater than 15 Å, even for single ions such as CL, NA and K. Finally, the last row contains heterogens for which the distribution of distances does not allow to indicate a clear proximity or remoteness with respect to the catalytic site. In fact they appear as part of protein sites which are not annotated as catalytic.

By merging the above-mentioned literature-based information with our analysis of the distances from catalytic residues, we derived the final classification into three groups reported in Table 10.

We encoded this information as a set of attributes (3*D*_8_) describing the presence of heterogen molecules in the 3D neighborhood of a residue. Following Table 10, this set includes three features counting the number of potentially catalytic, non-catalytic and uncertain heterogen molecules respectively.

**Table 10:** Heterogen analysis.

Class	Heterogens
**catalytic**	FE2, MN, CU1, MG, ZN3, ZN, HEM, HEG, HEC, SRM, MPD, MRD, FOK, PLP, P5P, PHS, OWQ, NO3, FS4, SF4, PVL, PYR, SEG, DHZ, FMT, HAD, CIT, ACN, PAC, ACT, 2PE, CNA, U5P, IKT, PGC, PGH, IMU, F6P, IMP, EEB, GLP, FBP, UD1, FCN, AZA, CRB, DHS, BME, ATP, ADP, GSH, FAD, FMN, SAM, AMP, NAD, GDP, GTP, GMP, MHF, NDP, NAG, NRI

**non-catalytic**	K, NA, NI, FE, CA, CL, SAC, FCY, PCA, MES, MAN

**uncertain**	PO4, PI, IPS, POP, SO4, SUL, GOL

Classification of the heterogens into three groups.

According to the catalytic residue definition given in [30], which guides the annotation of the residues as functional in the CSA database, residues which bind a substrate or a cofactor are not annotated as catalytic unless they are in some way directly involved in the catalytic process. This consideration can be particularly useful to discriminate among residues with a high catalytic propensity (e.g. CYS, HIS) that bind cofactors for structural reasons. We represented this information as an additional feature (3*D*_9_) encoding the presence of a bond between the target residue and a cofactor. We used a distance threshold of 3 Å for detecting bonds.

#### 3D Shapes

Geometric shapes extracted according to spatial considerations can be viewed as features characterizing a residue structural neighborhood. Planar shapes, for instance, can be viewed as substructures of the 3D space surrounding a residue and characterizing its interactions with the other residues.

We extracted planar shapes with two (segments) and three vertices (triangles) from the structural neighborhood of a residue. Each vertex corresponds to a residue that we labeled with its class type: charged (Ch), hydrophobic (Hy) or Polar (Po). This allowed us to reduce the sparseness of the whole set of shapes, thus increasing the likeliness of shape matches during the kernel evaluations. We represented the three-dimensional neighborhood of a residue as: (a) a cloud of points corresponding to the side-chain centroids of the residues, labeled with *Ch*, *Hy *or *Po *according to their class; (b) a graph where each pair of vertices in the cloud is connected by an edge if their distance is less than 5Å.

**Figure 10:**
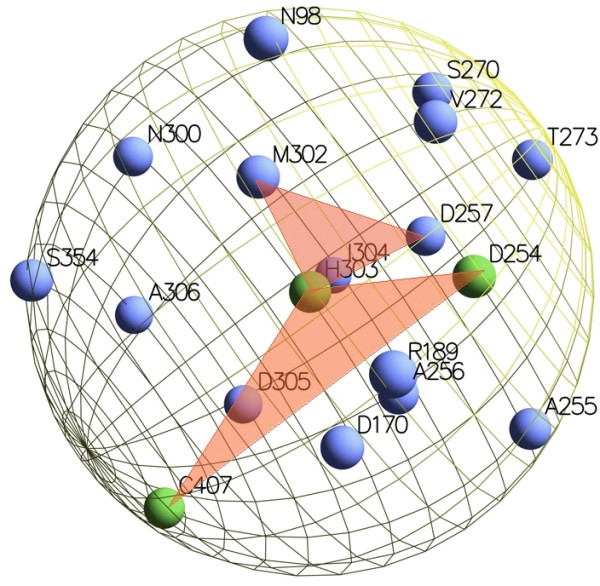
**Shapes**. Two examples of triangular shapes extracted from the HYS303 three-dimensional neighborhood.

From these two representations we extracted different sets of shapes to be used along with the 3D decomposition kernel: (1) shapes only composed of residues with conserved class, (2) shapes containing the target residue, and (3) shapes containing connected residues only (i.e. pairwise distances less than 5Å). We consider the class of a residue conserved when the sum of the profile entries corresponding to amino acids belonging to it is greater than 0.5. In Figure 10 two triangular shapes centered on the target residue HYS 303 are shown.

The 3D kernel measures the similarity between two residues in terms of the shapes which are shared between their respective 3D neighborhoods.

### Dataset Normalization

We normalized attribute values in the [-1,+1] range applying the following linear transformation: *value*' = 2.  - 1. While this implies a lower data sparsity with respect to a [0,1] normalization, preliminary experiments showed that it achieved better overall results. Missing values were managed by replacing categorical attributes with their modes and numerical attributes with their means, both computed from the distributions of observed values in the dataset.

## Authors' contributions

EC proposed the use of residue structural neighborhood, implemented the feature extraction tools and conducted all the experimental evaluation. AP suggested to model heterogen and ligand information and coordinated the whole study. Both authors contributed in writing the article. Both authors read and approved the final manuscript.

## Supplementary Material

Additional file 1 **Dataset used in the PW experiments **A .csv file (dataset.csv) containing the 23,635 examples identified by the tuple *(id, label, protein_PDB_Id, residue, chain, number)*, where *id *is an identifier to map the example in the 10 folds, *label *is +1 if the residue is annotated as catalytic and -1 otherwise, *protein_PDB_Id *is the PDB four letters identifier of the protein the residue belongs to, *residue *is the one letter amino acid code, *chain *is the chain of the protein in which the residue is located (being 'A' by default when no chain identifier is reported in the PDB file), *number *is the sequence number of the residue in the PDB file. Click here for file

Additional file 2 **10-fold details **A text file (10fold.txt) containing the list of proteins included in each fold of the cross-validation. Click here for file

Additional file 3 **Results **A .xls file (results.xls) reporting macro-averaged results of the best structure-based classifier. Click here for file

Additional file 4 **Predictions **A zip file (predfolds.zip) reporting predictions of the best structure-based classifier on the 10 folds. Click here for file

Additional file 5 **ROC and Recall/Precision curves **A pdf file (supplement.pdf) containing the ROC and Recall/Precision curves on the benchmark datasets. Click here for file
