# Supplementary material for: Automatic prediction of catalytic residues by modeling residue structural neighborhood
Source: BMC Bioinformatics. 2010 Mar 3;11:115. doi: 10.1186/1471-2105-11-115 (PMC2844391; doi:10.1186/1471-2105-11-115)
Supplement: Additional file 5 — ROC and Recall/Precision curves A pdf file (supplement.pdf) containing the ROC and Recall/Precision curves on the benchmark datasets. [file 1471-2105-11-115-S5.pdf]

Supplement of the article “Automatic Prediction of Catalytic Residues by Modeling Residue Structural Neighbourhood”

Elisa Cilia and Andrea Passerini
Dipartimento di Ingegneria e Scienza dell’Informazione
University of Trento - via Sommarive 14 - I38100 - Trento, Italy
{cilia,passerini}@disi.unitn.it

Curves

Figure 1 - Local ROC curves

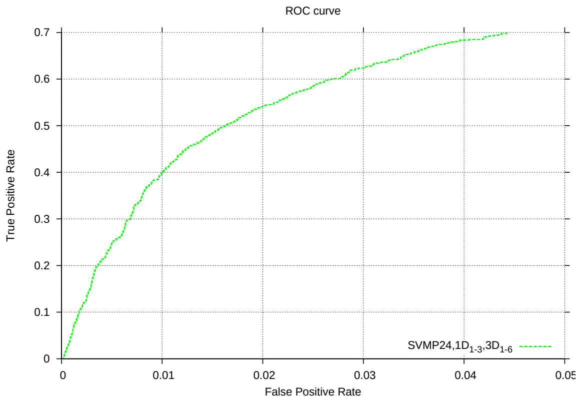

(a) Local ROC curve on the HA superfamily dataset

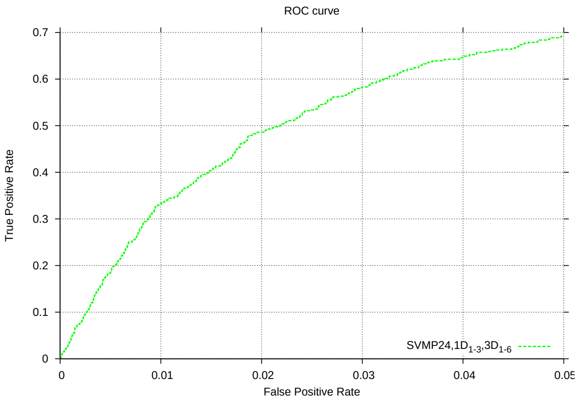

(b) Local ROC curve on the EF fold dataset

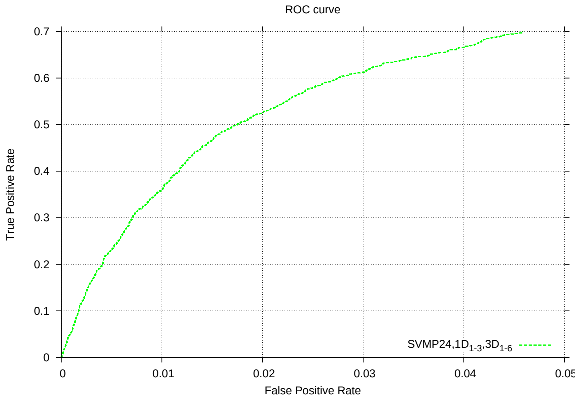

(c) Local ROC curve on the EF family dataset

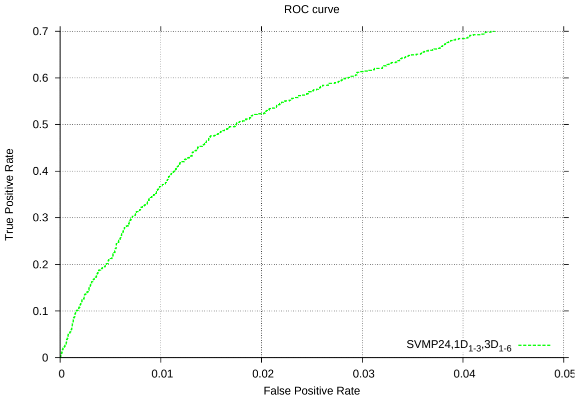

(d) Local ROC curve on the EF superfamily dataset

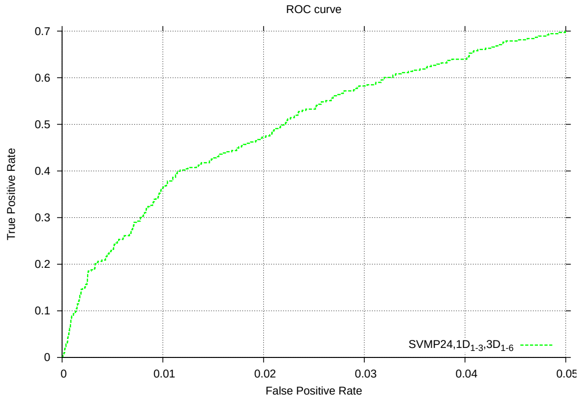

(e) Local ROC curve on the T-124 dataset

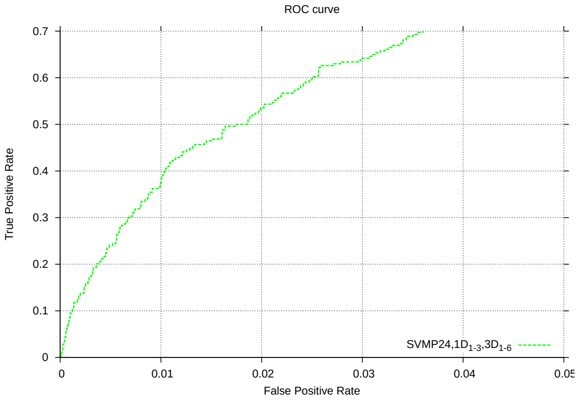

(f) Local ROC curve on the Petrova and Wu dataset

Figure 1: Local ROC curves of the predictions on different benchmark datasets.

Figure 2 - Global ROC curves

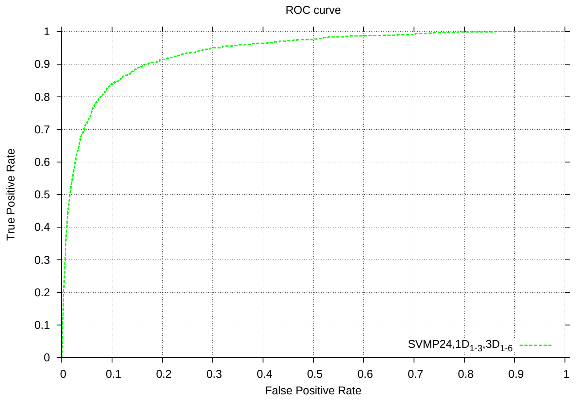

(a) Global ROC curve on the HA superfamily dataset

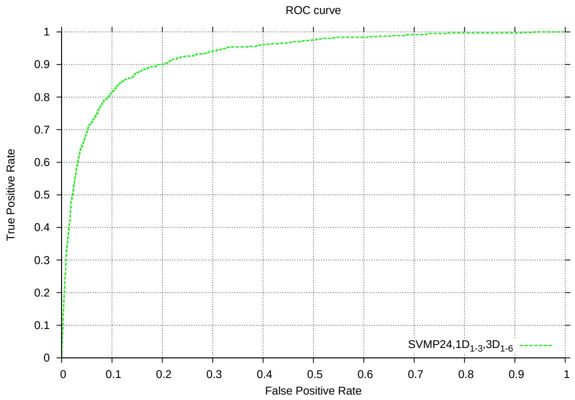

(b) Global ROC curve on the EF fold dataset

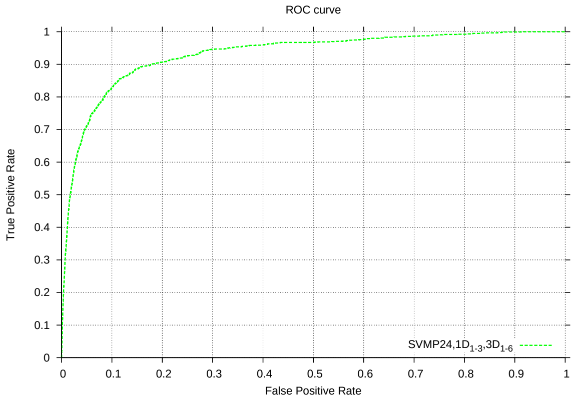

(c) Local ROC curve on the EF family dataset

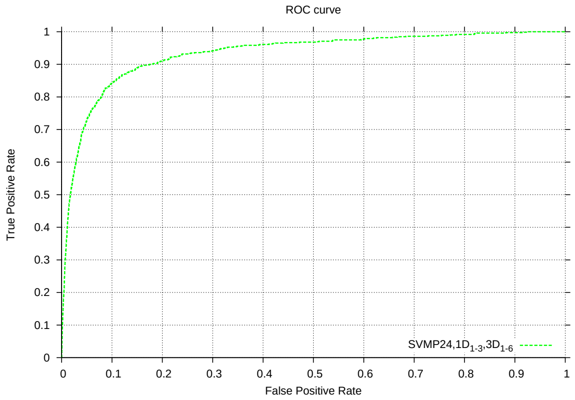

(d) Local ROC curve on the EF superfamily dataset

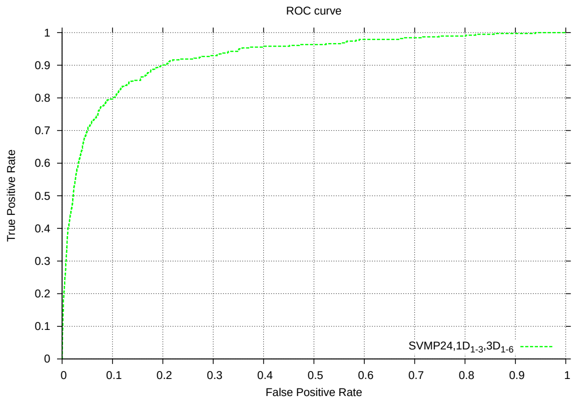

(e) Local ROC curve on the T-124 dataset

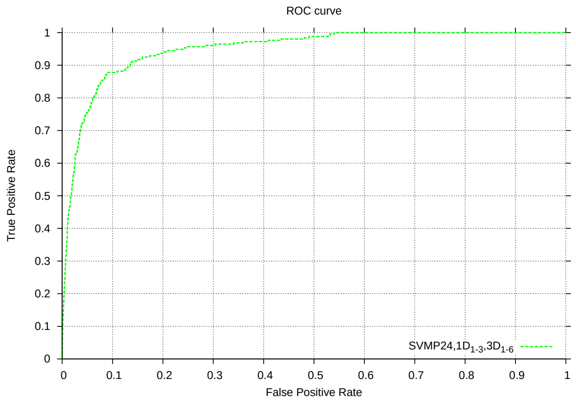

(f) Local ROC curve on the Petrova and Wu dataset

Figure 2: Global ROC curves of the predictions on different benchmark datasets.

Figure 3 - Recall/Precision curves

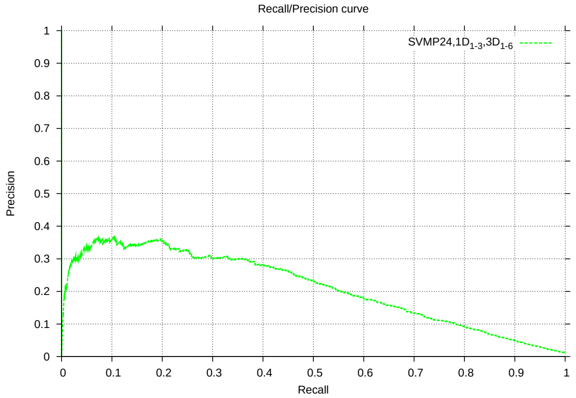

(a) Recall/Precision curve on the HA superfamily dataset

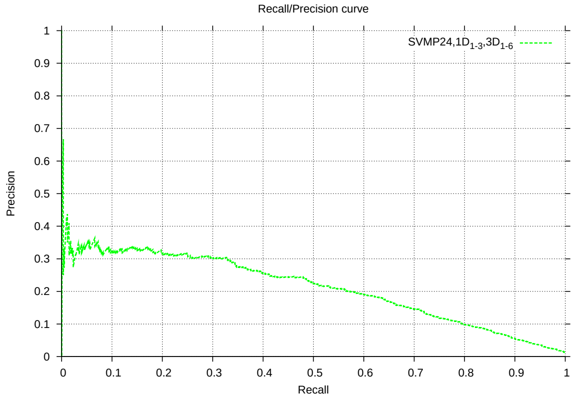

(b) Recall/Precision curve on the EF fold dataset

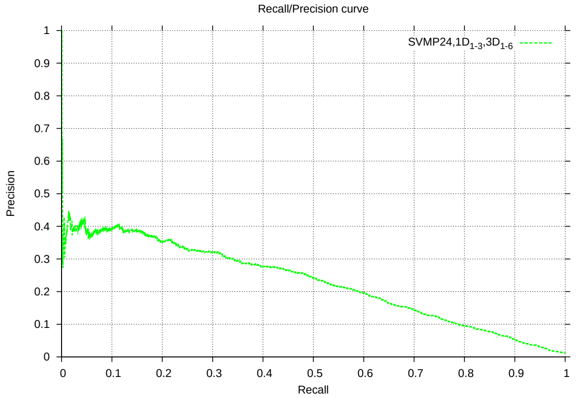

(c) Local ROC curve on the EF family dataset

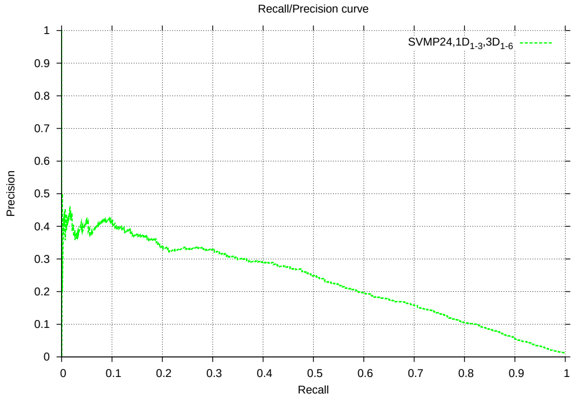

(d) Local ROC curve on the EF superfamily dataset

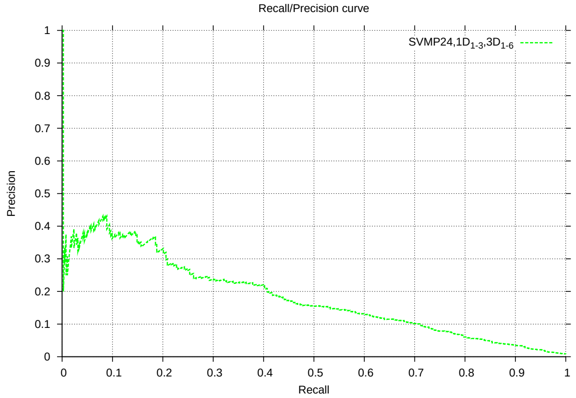

(e) Local ROC curve on the T-124 dataset

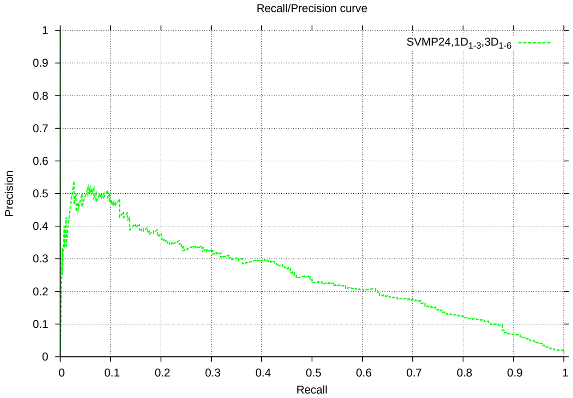

(f) Local ROC curve on the Petrova and Wu dataset

Figure 3: Recall/Precision curves of the predictions on different benchmark datasets.
